# Phosphorylation of MCPH1 isoforms during mitosis followed by isoform-specific degradation by APC/C-CDH1

**DOI:** 10.1096/fj.201801353R

**Published:** 2018-10-10

**Authors:** Stephanie K. Meyer, Michael Dunn, Daniel S. Vidler, Andrew Porter, Peter G. Blain, Paul A. Jowsey

**Affiliations:** * National Intitute for Health Research (NIHR) Health Protection Research Unit for Chemical and Radiation Threats and Hazards, Institute of Cellular Medicine, Newcastle University, Newcastle upon Tyne, United Kingdom; and; † Newcastle University Protein and Proteome Analysis, Newcastle University, Newcastle upon Tyne, United Kingdom

**Keywords:** cell cycle regulation, cyclin-dependent kinase-1, microcephalin-1, anaphase, promoting complex/cyclosome

## Abstract

Microcephalin-1 (MCPH1) exists as 2 isoforms that regulate cyclin-dependent kinase-1 activation and chromosome condensation during mitosis, with MCPH1 mutations causing primary microcephaly. MCPH1 is also a tumor suppressor protein, with roles in DNA damage repair/checkpoints. Despite these important roles, there is little information on the cellular regulation of MCPH1. We show that both MCPH1 isoforms are phosphorylated in a cyclin-dependent kinase-1–dependent manner in mitosis and identify several novel phosphorylation sites. Upon mitotic exit, MCPH1 isoforms were degraded by the anaphase-promoting complex/cyclosome–CDH1 E3 ligase complex. Anaphase-promoting complex/cyclosome–CDH1 target proteins generally have D-Box or KEN-Box degron sequences. We found that MCPH1 isoforms are degraded independently, with the long isoform degradation being D-Box dependent, whereas the short isoform was KEN-Box dependent. Our research identifies several novel mechanisms regulating MCPH1 and also highlights important issues with several commercial MCPH1 antibodies, with potential relevance to previously published data.—Meyer, S. K., Dunn, M., Vidler, D. S., Porter, A., Blain, P. G., Jowsey, P. A. Phosphorylation of MCPH1 isoforms during mitosis followed by isoform-specific degradation by APC/C-CDH1.

Mutations in microcephalin-1 (MCPH1) cause primary microcephaly, a rare genetic syndrome associated with a significantly reduced cerebral cortex and mental retardation (1, 2). Analysis of cells from affected individuals revealed a striking premature chromosome condensation (PCC) phenotype, characterized by an increased number of prophase-like cells (PLCs) with condensed chromatin and an intact nuclear membrane but lacking histone H3 Ser10 phosphorylation, a marker for mitotic cells (3–5). Interestingly, this phenotype was corrected by depletion of condensin II subunits (6). Subsequent studies revealed that MCPH1 was able to bind the condensin II complex directly and also to bind mitotic chromatin, effectively competing with and blocking the binding of condensin II (7, 8). Current models suggest that in the absence of MCPH1, condensin II is able to bind to chromatin and promote condensation before the onset of mitosis. Several studies have shown that cells lacking MCPH1 undergo chromatin condensation in the G2 phase of the cell cycle. A recent study found that inhibition of cyclin-dependent kinase-1 (CDK1) in MCPH1-deficient cells significantly reduced the proportion of cells with prematurely condensed chromatin (9). This finding supports other research showing that CDK1 is prematurely activated in cells lacking MCPH1, with CDK1 hypophosphorylation on Tyr15 apparent as soon as 2–4 h after release from S-phase synchronization (3). In addition, an elegant study using an MCPH1 knockout mouse revealed premature CDK1 activation in neuroprogenitor cells, due to mis-localization of checkpoint kinase 1 (CHK1) and activation of the CDC25B phosphatase (10). This scenario, in turn, caused spindle misalignment and a switch in the mode of cell division from symmetric to asymmetric, thus reducing the pool of neuroprogenitor cells in the developing brain.

In addition to being a key regulator of CDK1 activation and chromatin condensation during mitosis, MCPH1 is a tumor suppressor protein. Down-regulation and/or mutation of MCPH1 have been reported in breast, ovarian, colorectal, gastric, and colon cancer (11–14), and multiple studies have shown that MCPH1-deficient cells have DNA repair and checkpoint defects (13, 15, 16). MCPH1 is recruited to DNA double-strand breaks *via* phosphorylated histone H2AX and subsequently helps recruit BRCA2 (and thus RAD51), promoting homologous recombination repair (HRR) (17, 18). Cells lacking MCPH1 exhibit sensitivity to ionizing radiation and defects in the S-phase and G2/M checkpoints (15).

MCPH1 contains an N-terminal BRCT (BRCA1 C-terminal) domain and 2 C-terminal BRCT domains. The latter are required for recruitment to DNA damage foci *via* binding to phosphorylated histone H2AX (Ser139), whereas the N-terminal domain is believed to be involved in chromatin binding and the regulation of chromosome condensation. MCPH1 exists as 2 major isoforms: a long isoform (MCPH1-L) containing all 3 BRCT domains and a short isoform (MCPH1-S) produced *via* a splicing-inserted stop codon, which eliminates both C-terminal BRCT domains (19). Studies suggest that both isoforms contribute to chromosome condensation, whereas the DNA damage roles of MCPH1 are believed to predominantly involve the long isoform, highlighting the importance of the C-terminal BRCT domain pair. Although other isoforms have been reported, the biologic significance of these isoforms is less clear.

Despite the important cellular roles of both MCPH1 isoforms, little information is available regarding their cellular regulation. An earlier study suggested that MCPH1 was not cell cycle regulated, with no apparent change in abundance or gel migration pattern in cell synchronization experiments (17). More recently, Drosophila MCPH1 was shown to be regulated by the anaphase-promoting complex/cyclosome (APC/C), although the same study suggested that human MCPH1 was not similarly regulated (20). The APC/C is an E3 ubiquitin ligase that drives the metaphase–anaphase transition and is activated by binding to either CDC20 or CDH1 (21, 22). The former targets proteins during the metaphase–anaphase transition, before being replaced by CDH1 during late mitosis and G1, changing the substrate specificity of the APC/C. CDC20 and CDH1 generally recognize target proteins *via* specific degron sequences, called D-Box (minimal consensus of RxxL) and KEN-Box. A recent study showed that the long isoform of human MCPH1 could be targeted by the APC/C–CDH1 complex, although the kinetics of apparent degradation did not align with APC/C–CDH1 activation, with decreased MCPH1 levels observed before cyclin B1 degradation (a CDC20 target) (23). These findings are likely due to the particular antibody used and are addressed as part of the present study.

The present study aimed to clarify some of the conflicting findings in the literature regarding the cell cycle–dependent regulation of MCPH1. After thorough characterization of commercial antibodies, we were able to clearly show CDK1-dependent phosphorylation of both MCPH1 isoforms during mitosis, followed by APC/C–CDH1–dependent degradation in the G1 phase. Our data show that 1 commercial antibody is blocked from binding phosphorylated MCPH1, with potential relevance to previously published studies. Using mass spectrometry, we identified several novel phosphorylation sites in MCPH1 purified from mitotic cells, including 2 that were within/near a functionally relevant D-Box. Interestingly, although both MCPH1 isoforms were regulated by APC/C–CDH1, this regulation involved different degron sequences. Two functionally important degron sequences were identified within MCPH1-L. The first sequence is a C-terminal D-Box (amino acids 752–755), which is necessary for CDH1-dependent degradation of MCPH1-L. The second sequence is a KEN-Box sequence (amino acids 599–601), the mutation of which markedly reduced the interaction between MCPH1-L and CDH1 but had no effect on CDH1-mediated degradation of MCPH1-L. The functional significance of the KEN-Box–dependent interaction between MCPH1-L and CDH1 remains to be elucidated. Interestingly, this same KEN-Box was found to control the CDH1-dependent degradation of the MCPH1-S isoform.

## MATERIALS AND METHODS

### Cell lines, chemicals, and treatments

HEK293, A549, U2OS, and HeLa cells were obtained from Public Health England (PHE) Culture Collections (Salisbury, United Kingdom). HeLa Tet-On cells were obtained from Thermo Fisher Scientific (Waltham, MA, USA). All cells were maintained as exponentially growing cultures in DMEM supplemented with 10% fetal bovine serum and 2 mM l-glutamine. Nocodazole, l-mimosine, cycloheximide, and tetracycline were obtained from MilliporeSigma (Burlington, MA, USA), RO3306 (CDK1 inhibitor) from Tocris Bioscience (Minneapolis, MN, USA), and ProTAME (APC/C inhibitor) from Bio-Techne (Minneapolis, MN, USA). Stock solutions of all compounds were prepared in DMSO, except for l-mimosine, which was dissolved in 20 mM HEPES (pH 7). Compounds were added to cells at the indicated concentrations (as noted in the figure legends), with DMSO levels being maintained at <0.2% (v/v).

### Plasmids, mutagenesis, small interfering RNA, and transfections

A plasmid encoding human MCPH1-L (full length or long isoform) was kindly provided by Professor Shiaw-Yih Lin (MD Anderson Cancer Center, University of Texas, USA). This plasmid was used as a template to subclone MCPH1-L into an expression plasmid with an N-terminal green fluorescent protein (GFP) tag (PS100048; OriGene Technologies, Rockville, MD, USA). Plasmids encoding human CDC20 and CDH1 were obtained from Dr. Jacques Bertoglio (Institut Gustave Roussy, Villejuif, France) and used as a template for subcloning of each into an expression plasmid with an N-terminal FLAG tag (PS100014; OriGene Technologies). Plasmids encoding tetracycline-inducible GFP–MCPH1-L and GFP–MCPH1-S were kindly provided by Professor Andrew Jackson (Edinburgh University, Edinburgh, United Kingdom). Fragments of MCPH1 and CDH1 were generated by the introduction of stop codons using site-directed mutagenesis, with the same technique used to mutate specific D- and KEN-boxes in MCPH1. Site-directed mutagenesis was performed by using either Phusion Hot Start II High-Fidelity DNA Polymerase (Thermo Fisher Scientific) or a QuikChange XL II kit (Stratagene, San Diego, CA, USA), according to the manufacturer’s instructions. PCR reactions were subject to Dpn1 digestion before transformation into NEB 10-β competent *Escherichia coli* cells (New England Biolabs, Ipswich, MA, USA) and antibiotic selection on Luria broth (LB) agar plates. Selected clones were analyzed by using Sanger sequencing to confirm mutated DNA sequences.

For protein interaction studies, HEK293 cells were transfected by using calcium phosphate precipitation. Plasmids were transfected into HeLa cells using Lipofectamine 2000 (Thermo Fisher Scientific), with slight modifications to the manufacturer’s recommended instructions. For cells plated in 6 cm dishes, 3.5 μg DNA and 4 μl Lipofectamine 2000 were used (both added to 150 μl serum-free DMEM in separate tubes, before combining in a single tube). For cotransfection of GFP–MCPH1 (and mutants) with FLAG/FLAG–CDC20/FLAG–CDH1 in HeLa Tet-On cells, 2.5 μg GFP–MCPH1 and 1 μg FLAG–CDH1 per 6 cm dish were used, and GFP–MCPH1 expression was induced by adding tetracycline 10 ng/ml.

For small interfering RNA (siRNA), the following sequences were used to target MCPH1: AAAGGAAGTTGGAAGGATCCA (Qiagen, Germantown, MD, USA) and SMARTpool ON-TARGETplus (L-008447-00-0005; Dharmacon, Lafayette, CO, USA). To target CDH1, SMARTpool ON-TARGETplus siRNA was used (L-015377-00-0005; Dharmacon). RNA was transfected into cells (20 nM final concentration) by using RNAiMAX (Thermo Fisher Scientific) for 48–72 h.

### Immunoprecipitation/pull-down, Western blotting, and antibodies

To investigate the interaction between MCPH1 and CDH1, a pull-down approach was used. GFP–MCPH1 was immunoprecipitated from HEK293 cells before incubation with cell extracts containing FLAG-tagged CDH1 (or CDH1 fragments). For immunoprecipitation of GFP–MCPH1, cells were washed in cold PBS before lysis in NETN buffer (50 mM Tris pH 7.6, 150 mM NaCl, 1 mM EDTA, and 1% NP-40) containing protease and phosphatase inhibitors (MS-SAFE cocktail; MilliporeSigma). After incubation at 4°C for 15 min, lysates were cleared by centrifugation and supernatants isolated. Cell lysates were incubated with GFP-Trap (Chromotek, Planegg-Martinsried, Germany) to purify GFP-tagged proteins for 2 h at 4°C before washing 2 times in TBST (50 mM Tris pH 7.6, 150 mM NaCl, and 0.2% Tween-20), followed by a wash in NETN. After removal of this supernatant, cell extracts containing FLAG–CDH1 (prepared in NETN buffer) were added to GFP–MCPH1 immobilized on GFP–TRAP beads. After incubation for 3 h at 4°C, the beads were washed 3 times in TBST and resuspended in an equal volume of 2× LDS Sample Buffer (Thermo Fisher Scientific) containing 5% 2-ME and heated to 70°C for 10 min. Samples were analyzed by Western blotting using 3–8% Tris acetate gels (Thermo Fisher Scientific) before transfer to nitrocellulose using an iBlot2 machine (Thermo Fisher Scientific) and antibody incubation (discussed later).

For Western blotting of whole cell extracts, cells were lysed directly into 2× LDS Sample Buffer containing 2.5% 2-ME, heated to 70°C for 10 min, and sonicated to shear genomic DNA. Protein electrophoresis was performed by using either 3–8% Tris acetate or 4–12% bis Tris gels (Thermo Fisher Scientific) before transferring to nitrocellulose by using an iBlot2 machine. Membranes were blocked in either 5% dried skimmed milk/TBST or 2.5% bovine serum albumin/TBST (depending on the primary antibody used) for 1 h before overnight incubation in blocking buffer containing the following primary antibodies: MCPH1 (11962-1-AP; Proteintech, Rosemont, IL, USA), MCPH1 (Ab2612; Abcam, Cambridge, United Kingdom), CDH1/FZR1 (NBP-54465; Novus Biologicals, Littleton, CO, USA), and mitotic (phospho) protein monoclonal-2 (MilliporeSigma). In addition, the following primary antibodies from Cell Signaling Technology (Danvers, MA, USA) were also used: GFP (2956), glyceraldehyde-3-phosphate dehydrogenase (2118), histone [^3^H] phospho-Serine 10 (9701), DYKDDDDK/FLAG tag (2368), cyclin B1 (4135), phospho-cdc2 Tyr15 (9111), and MCPH1 (4120). After incubation with the relevant horseradish peroxidase–conjugated secondary antibody, Western blots were visualized with ECL Prime (GE Healthcare, Chicago, IL, USA) and images captured by using a Syngene (Cambridge, United Kingdom) G:Box gel documentation system.

### Mass spectrometry

HEK293 cells were transfected with GFP–MCPH1 for 24 h using calcium phosphate before treatment with nocodazole for 20 h. Mitotic cells were isolated by using the shake-off procedure, lysed in NETN buffer (as previously described), and GFP–MCPH1 immunoprecipitated and digested by using the iST GFP-TRAP kit from Chromotek, according to the manufacturer’s instructions. Dried peptides were reconstituted in 25 μl of 95:5 water:acetonitrile 0.1% formic acid. The tryptic digest was analyzed by using data-dependent techniques on a TripleTOF 5600+ high-resolution quadrupole time-of-flight mass spectrometer (Sciex, Framingham, MA, USA) equipped with a DuoSpray Ion Source operated in positive electrospray mode, coupled to an Eksigent NanoLC 420 system (Sciex). Analyst TF v.1.7.1 (Sciex) was used for instrument control and data acquisition. Chromatographic separation was achieved by gradient elution over 95 min, with an ACE C_18_ capillary liquid chromatography column (100 mm × 300 µm × 3 µm; Hichrom, Theale, United Kingdom) fitted with a 0.25 µm column saver precolumn filter, using 0.1% formic acid in water and 0.1% formic acid in acetonitrile as the mobile phase, at a flow rate of 5 µl/min. A 5 µl injection was used. Data-dependent analysis comprised a 250 ms survey scan, in which the 20 most intense ions were selected for subsequent automated tandem mass spectrometry (MS/MS) analysis, with each MS/MS event consisting of a 50 ms scan. Ions were isolated by using a quadrupole resolution of 0.7 Da and fragmented in the collision cell by using collision energy ramped from 15 to 45 eV within the 50 ms accumulation time. The data-dependent MS/MS data were processed by using ProteinPilot software version 4.5 (Sciex). Data were searched against the uniprot_sprot_can+iso database, with phosphorylation emphasis (*https://www.uniprot.org/*).

### Premature chromosome condensation

U2OS cells were cotransfected with siRNA targeting MCPH1, or nontargeting control, along with GFP or GFP-tagged MCPH1 (siRNA resistant) using Lipofectamine 2000. After 72 h, cells were fixed with 3.7% formaldehyde, washed in PBS, and then incubated in PBS/0.2% Triton X-100 containing DAPI for 5 min. Cells were then washed in PBS and a coverslip applied using ProLong Gold antifade mounting medium (Thermo Fisher Scientific). Cells were analyzed by using fluorescence microscopy and the percentage of prophase-like cells counted (*i.e.*, cells with condensed chromatin but intact nuclear membrane). The significance of the observed changes in PCC was investigated by using a paired *t* test, with values of *P* < 0.05 denoted by an asterisk.

## RESULTS

### MCPH1 isoforms are regulated in a cell cycle–dependent manner

**Figure 1:**
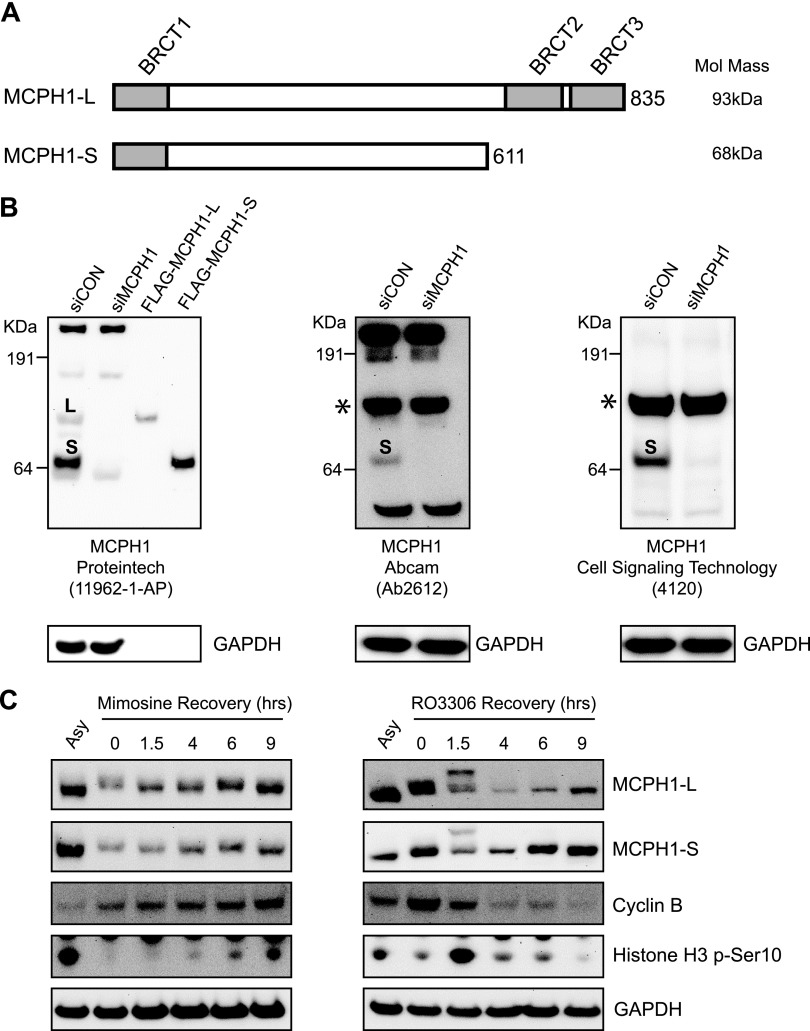
MCPH1 isoforms are regulated in a cell cycle–dependent manner. *A*) Schematic diagram showing the domain structure and molecular mass of MCPH1-L and MCPH1-S. *B*) U2OS cells were transfected with control siRNA or siRNA targeting MCPH1 for 48 h before Western blotting with the indicated commercial antibodies. In the left-hand panel, samples of purified FLAG–MCPH1-L and FLAG–MCPH1-S were included for comparison with endogenous MCPH1 protein bands. “L” and “S” indicate bands corresponding to the long and short MCPH1 isoforms, respectively. *C*) HeLa cells were synchronized in G1 with 500 μM l-mimosine (20 h) or in G2 with 9 μM RO3306 (20 h) before release into fresh medium for the indicated time points and Western blot analysis.

MCPH1 exists as 2 major isoforms, as schematically represented in **Fig. 1*A***, with MCPH1-L (835 aa) consisting of 1 N-terminal and 2 C-terminal BRCT domains. MCPH1-S (611 aa) lacks the pair of C-terminal BRCT domains. It is currently not clear whether MCPH1 is regulated in a cell cycle–dependent manner, with conflicting reports being published. Our goal was to investigate both major MCPH1 isoforms, and we initially validated several commercial antibodies by Western blotting of control and MCPH1-depleted cells. Only 1 antibody was identified that was able to robustly detect both MCPH1 isoforms in our experimental conditions. As shown in Fig. 1*B* (left-hand panel), the antibody from Proteintech detected 2 bands at the predicted MW for MCPH1-L and MCPH1-S in cell extracts, with both bands lost in cells transfected with MCPH1 siRNA. This antibody was also able to detect immunoprecipitated FLAG-tagged MCPH1-L and MCPH1-S, which both aligned well with the bands for endogenous isoforms. In contrast, 2 other commercial antibodies (from Abcam and Cell Signaling Technology) detected a strong nonspecific signal at the approximate MW of MCPH1-L (highlighted by an asterisk in Fig. 1*B*, middle and right-hand panels). Importantly, this band was not decreased after MCPH1 siRNA. Both of these antibodies were able to detect MCPH1-S.

To investigate whether MCPH1 protein levels changed through the cell cycle, HeLa cells were synchronized in G1 by using l-mimosine or in G2 by using RO3306 (a CDK1 inhibitor). After release into fresh medium and progression through the cell cycle, MCPH1 isoforms were analyzed at specific time points postrelease using Western blotting. As shown in Fig. 1*C* (left-hand panel), the levels of MCPH1-L and MCPH1-S were both decreased after synchronization in G1 (compare mimosine-treated cells in lane 2 with asynchronous cells in lane 1). After release from the G1 block, the levels of both MCPH1 isoforms gradually increased as cells progressed toward/through the S phase and toward G2/mitosis (as shown by the increase in cyclin B and increased phosphorylation of histone H3 serine 10 after 9 h release). After release from a G2 block (Fig. 1*C*, right-hand panel), cells rapidly entered mitosis (marked increase in histone H3 serine 10 phosphorylation 1.5 h post-release). At this same time point, MCPH1-L and MCPH1-S both underwent a gel mobility shift, consistent with protein phosphorylation (phosphatase treatment of cell extracts eliminated this mobility shift; data not shown). At 4 h post-release, levels of MCPH1-L and MCPH1-S were both decreased, before beginning to increase after 6 and 9 h. These data suggest that both MCPH1 isoforms are regulated in a cell cycle–dependent manner, with phosphorylation during mitosis and decreased protein levels as cells exit mitosis and enter the G1 phase of the cell cycle.

### MCPH1 isoforms are phosphorylated during mitosis

**Figure 2:**
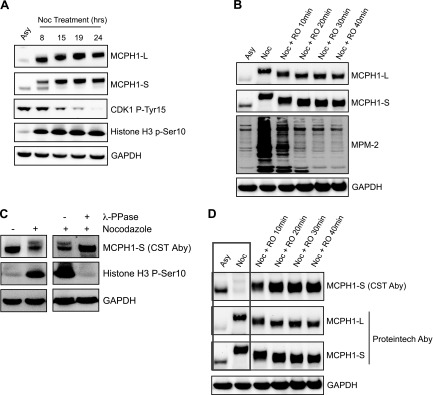
MCPH1 isoforms are phosphorylated during mitosis. *A*) U2OS cells were treated with 100 ng/ml nocodazole for the indicated time points before Western blot analysis. Asynchronous cells (Asy) were also analyzed. *B*) U2OS cells were synchronized in prometaphase by treatment with 100 ng/ml nocodazole (20 h). RO3306 (9 μM) was then added to the culture medium for the indicated time points before Western blot analysis. *C*) U2OS cells were treated with nocodazole (100 ng/ml) for 20 h before Western blot analysis. In the right-hand panel, nocodazole-treated cells were lysed in native lysis buffer before addition (or not) of λ-phosphatase for 20 min at 30°C. Samples were then analyzed by using Western blotting. *D*) Samples from *B* were reanalyzed by Western blotting to allow comparison between 2 different MCPH1 antibodies (Cell Signaling Technology/CST *vs.* Proteintech). Aby, antibody.

Figure 1*C* shows that as cells are released from the G2 block and enter mitosis, both MCPH1 isoforms undergo a gel mobility shift, consistent with protein phosphorylation. The mobility shift was reversed by λ phosphatase treatment of mitotic cell extracts (data not shown). To further investigate the potential regulation of MCPH1 isoforms during mitosis, a nocodazole time-course treatment was performed to synchronize cells in prometaphase. Nocodazole disrupts mitotic spindles and activates the spindle assembly checkpoint, which prevents progression to anaphase *via* inhibition of the APC/C. As shown in **Fig. 2*A***, MCPH1-L and MCPH1-S both undergo a gel mobility shift as cells are blocked in mitosis, as well as an increase in protein abundance. The MCPH1 mobility shift correlated well with the activation of CDK1, as indicated by hypophosphorylation of CDK1 Tyr15. Similar results were obtained with Taxol (MilliporeSigma) (data not shown). CDK1 is highly active in nocodazole-treated cells, and we used a specific CDK1 inhibitor (RO3306) to investigate whether the observed MCPH1 phosphorylation was CDK1 dependent. As shown in Fig. 2*B*, addition of RO3306 to nocodazole-treated cells caused a rapid loss (apparent at 10 min) of the slower migrating band (*i.e.*, loss of phosphorylated form) of MCPH1-L and MCPH1-S. To confirm CDK1 inhibition by RO3306, extracts were analyzed by using the mitotic (phospho) protein monoclonal-2 antibody, which recognizes multiple CDK1 target proteins phosphorylated in mitosis. Consistent with this finding, a very strong signal was observed after nocodazole treatment, which was lost after CDK1 inhibition. At longer time points after RO3306 treatment, MCPH1 levels decrease (data not shown).

Similar studies were also performed by using a different commercial antibody (from Cell Signaling Technology/CST), which detects only MCPH1-S under our experimental conditions. As shown in Fig. 2*C*, nocodazole treatment caused an apparent decrease in MCPH1-S. However, this effect was reversed by phosphatase treatment of cell extracts, strongly suggesting that the antibody recognition of MCPH1-S is being adversely affected by phosphorylation. To further investigate this theory, we reanalyzed the samples from Fig. 2*B* and compared results with findings with the Proteintech antibody. The Cell Signaling Technology antibody exhibited decreased recognition of MCPH1-S in nocodazole-treated cells (Fig. 2*D*), which was rapidly reversed by treatment with the CDK1 inhibitor (*i.e.*, as MCPH1 is being dephosphorylated). Given that this change occurs within 10 min, it is very unlikely to be due to an actual change in protein abundance. Along with the data in Fig. 2*C*, we conclude that the Cell Signaling Technology antibody recognizes an epitope within MCPH1 that is phosphorylated during mitosis and this action impedes antibody binding.

Given the marked gel migration shift in MCPH1-L and MCPH1-S after nocodazole, it is likely that MCPH1 is heavily phosphorylated. To identify sites of phosphorylation, GFP–MCPH1-L was purified from mitotic cells and analyzed by using mass spectrometry. Multiple phosphorylated peptides were identified, as summarized in **Table 1**. Seven of these have been published previously, and 4 were novel. CDK1 phosphorylates the minimal consensus sequence S/T-P. Several of the identified sites conform to this sequence, although additional studies will be required to verify MCPH1 sites that are directly phosphorylated by CDK1. Interestingly, 2 of the new sites (Thr754 and Ser769) are within/near a potential D-Box degron site and were investigated further later in this study.

**TABLE 1:** Phosphorylation sites identified in GFP–MCPH1-L from nocodazole-treated cells

Peptide	Amino acid	Previously published
TFTTQLVDMGAK	T27	No
KLEGSINDIK	S254	Yes
ANNIHSSPSFTHLDK	S276	Yes
ANNIHSSPSFTHLDK	S277	Yes
ANNIHSSPSFTHLDK	S279	Yes
YRLSPTLSSTK	S333	Yes
YSENLPPESQLPSSPAQLSCR	S437	Yes
YSENLPPESQLPSSPAQLSCR	S438	Yes
GTLFADQPVMFVSPASSPPVAK	T754	No
GTLFADQPVMFVSPASSPPVAK	S769	No
QASIVIGPYSGK	S801	No

HEK293 cells were transfected with GFP–MCPH1-L for 24 h before nocodazole treatment (100 ng/ml, 20 h). GFP–MCPH1 was purified from cells after mitotic shake-off and digested with trypsin/pronase. Peptides were analyzed by mass spectrometry using a Sciex TripleTOF 5600. The phosphorylated peptides are listed, with the site of phosphorylation underlined.

Together, these data show that MCPH1-L and MCPH1-S are phosphorylated in a CDK1-dependent manner during mitosis.

### MCPH1 isoforms are regulated by APC/C–CDH1

**Figure 3:**
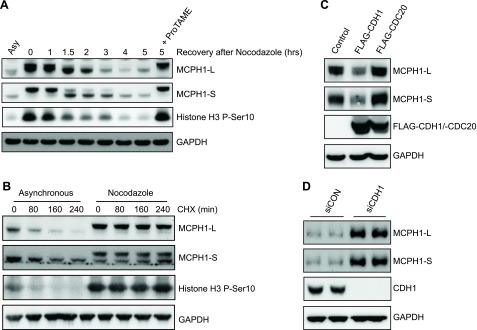
MCPH1 isoforms are regulated by APC/C–CDH1. *A*) A549 cells were treated with 100 ng/ml nocodazole for 20 h before release into fresh culture medium for the indicated time points. ProTAME (12 μM) was also included in one 5 h release sample before Western blot analysis. *B*) Asynchronous and nocodazole-synchronized cells were treated with 50 μg/ml cycloheximide (CHX) for the indicated time points to block protein synthesis before Western blotting. *C*) A549 cells were transfected with FLAG empty vector, FLAG–CDH1, or FLAG–CDC20 for 48 h before Western blot analysis. *D*) A549 cells were transfected with control siRNA or CDH1 siRNA for 72 h before Western blot analysis.

The accumulation of MCPH1 isoforms after cells are blocked in prometaphase (Fig. 2*A*) and the decrease in MCPH1 protein levels as cells exit mitosis (Fig. 1*C*) are consistent with MCPH1 being a target of the APC/C complex. To verify this theory, cells were released from a nocodazole block and MCPH1 isoforms analyzed by using Western blotting. As shown in **Fig. 3*A***, there was a loss of both MCPH1-L and MCPH1-S 2–5 h after nocodazole release, which was blocked by treatment of cells with an APC/C inhibitor (ProTAME). To further verify the role of APC/C in MCPH1 regulation, a protein half-life study was performed. Cycloheximide treatment of asynchronous cells caused a time-dependent decrease in MCPH1-L and MCPH1-S (Fig. 3*B*). In contrast, cycloheximide treatment of cells synchronized in mitosis (by nocodazole treatment) showed no decrease in the levels of either MCPH1 isoform. Importantly, APC/C is inhibited in nocodazole-treated cells due to activation of the spindle assembly checkpoint. APC/C is activated by binding to either CDC20 (promoting metaphase–anaphase transition) or CDH1 (promoting mitotic exit/G1 progression) activator proteins. The kinetics of MCPH1 isoform degradation are consistent with APC/C–CDH1 activity. To investigate this theory, CDH1 or CDC20 were overexpressed in cells and levels of MCPH1 investigated. As shown in Fig. 3*C*, CDH1 caused a marked decrease in both MCPH1-L and MCPH1-S, whereas CDC20 did not. To further validate these findings, CDH1 was depleted from cells using siRNA, causing a marked increase in both MCPH1 isoforms (Fig. 3*D*). Together, these studies show that MCPH1-L and MCPH1-S are both degraded upon mitotic exit in an APC/C–CDH1–dependent manner.

### Characterization of the MCPH1–CDH1 interaction

**Figure 4:**
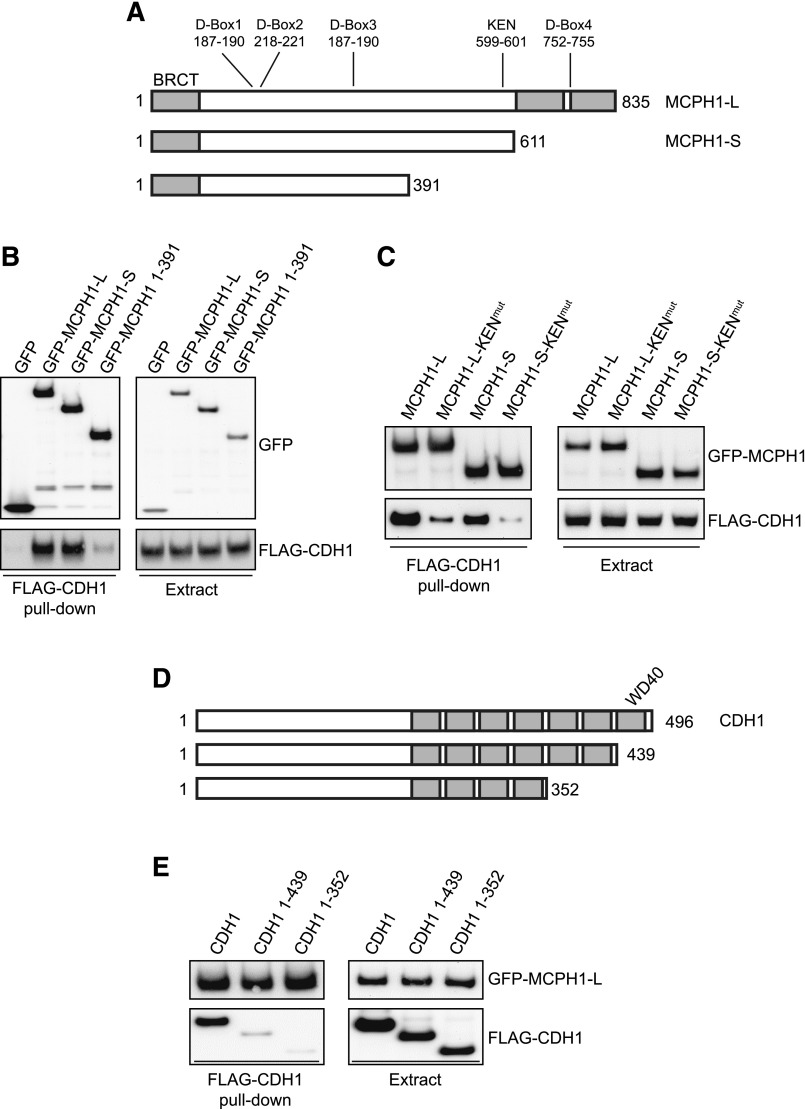
Characterization of the MCPH1–CDH1 interaction. *A*) Schematic diagram of MCPH1 fragments to be used in CDH1 pull-down studies. The sites of potential D-Box and KEN-Box degron sequences are indicated. *B*) GFP or GFP–MCPH1 variants were transfected into HEK293 cells for 24 h before purification and incubation with FLAG–CDH1 cell extracts (from transfected HEK293 cells). The ability of CDH1 to bind MCPH1-L was investigated by using Western blotting. *C*) The KEN-Box sequence was mutated to “AAA” in GFP–MCPH1-L and GFP–MCPH1-S before performing the CDH1 pull-down experiment as described in *B*. *D*) Schematic diagram of CDH1 fragments to be used in pull-down studies with GFP–MCPH1-L. *E*) FLAG–CDH1 (or CDH1 fragment) pull-down was performed as in *B*.

We next investigated the potential interaction between MCPH1 and CDH1. Given that CDH1 targets MCPH1 for degradation in cells, we chose to use a pull-down approach for these studies, rather than coexpression/purification of both proteins. HEK293 cells were transfected with GFP control or one of the GFP-tagged constructs depicted in **Fig. 4*A***. GFP/GFP–MCPH1 was then purified from cells before incubation with a FLAG–CDH1 cell extract. The potential interaction between MCPH1 and CDH1 was then investigated by using Western blotting. As shown in Fig. 4*B*, both MCPH1-L and MCPH1-S were able to bind CDH1, whereas GFP and MCPH1 1–391 were not. These data would suggest that a region of MCPH1 between 391 and 611 is important for the interaction with CDH1. CDH1 generally recognizes target proteins *via* D-Box or KEN-Box degron sequences. Analysis of the MCPH1 amino acid sequence revealed several potential degron sequences, including a potential KEN-Box from 599 to 601. Each of the KEN-box residues were mutated to alanine (KEN^mut^) in MCPH1-L and MCPH1-S and the CDH1 pull-down performed. Interestingly, mutation of the KEN-Box significantly reduced the binding of both MCPH1 isoforms to CDH1 (Fig. 4*C*).

We also investigated the regions of CDH1 that were important for binding to MCPH1, with CDH1 containing several WD40 domains that mediate protein–protein interactions. The FLAG-tagged CDH1 constructs depicted in Fig. 4*D* were incubated with purified GFP–MCPH1-L before Western blotting. As shown in Fig. 4*E*, loss of the extreme C-terminal WD40 domain markedly reduced the interaction between MCPH1-L and CDH1.

Together, these data suggest that the KEN-Box in both MCPH1 isoforms contributes to CDH1 binding and that the seventh WD40 domain in CDH1 is important for binding to MCPH1.

### Isoform-specific regulation of MCPH1 by APC/C–CDH1

**Figure 5:**
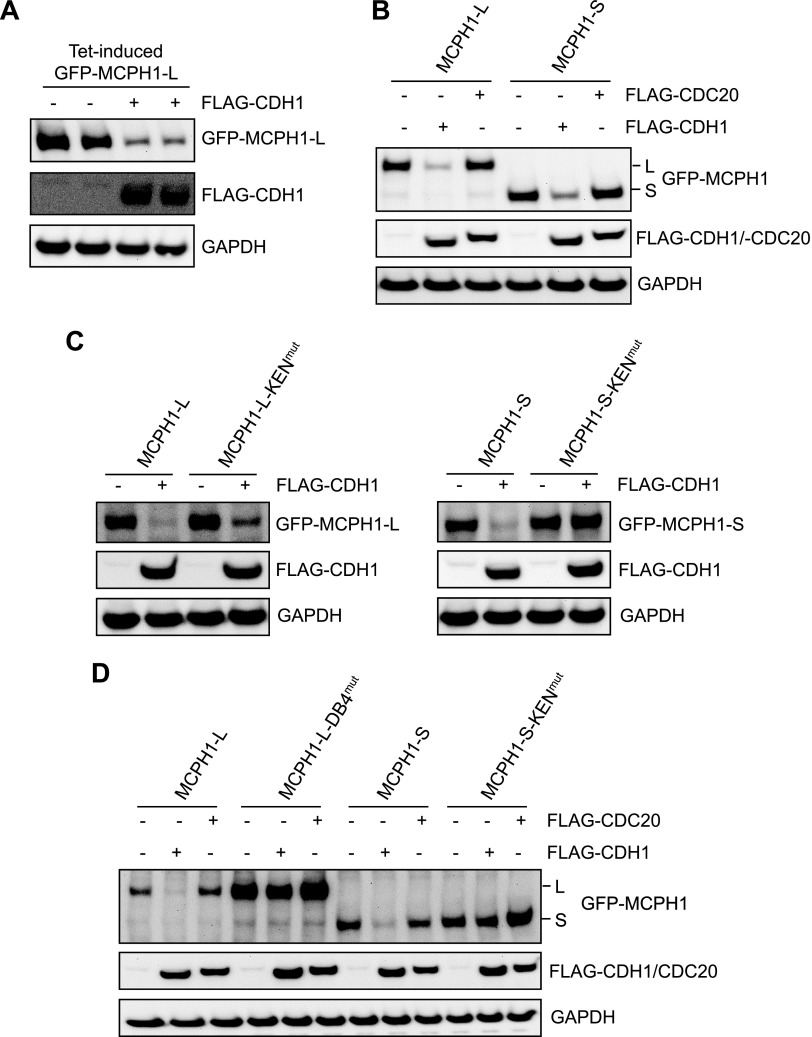
Isoform-specific regulation of MCPH1 by APC/C–CDH1. *A*) HeLa Tet-On cells were cotransfected with GFP–MCPH1-L and either FLAG empty vector or FLAG–CDH1. GFP–MCPH1-L expression was induced by the addition of tetracycline 10 ng/ml. After 24 h, cells were lysed and samples analyzed by using Western blotting. *B*) HeLa Tet-On cells were cotransfected with GFP–MCPH1-L or GFP–MCPH1-S, along with either FLAG empty vector or FLAG–CDC20 or FLAG–CDH1. GFP–MCPH1 expression was induced and samples processed as in *A*. *C*, *D*) HeLa Tet-ON cells were cotransfected with the indicated plasmids and processed as in *A*.

APC/C–CDH1 target proteins generally contain either D-Box or KEN-Box degron sequences. Analysis of the MCPH1 amino acid sequence revealed 4 potential D-Boxes and 1 potential KEN-Box. Given that we have shown that both MCPH1-L and MCPH1-S bind CDH1 in a KEN-Box–dependent manner, we hypothesized that the CDH1-dependent degradation of both isoforms would be similarly reliant on this KEN-Box. We established an experimental system that allowed cotransfection of GFP–MCPH1 with either FLAG empty vector or FLAG–CDH1. We used an inducible GFP–MCPH1 plasmid to control MCPH1 expression and showed that cotransfection with FLAG–CDH1 caused a marked decrease in GFP–MCPH1 compared with cotransfection with FLAG vector (**Fig. 5*A***). We validated this system further by showing that both GFP–MCPH1-L and GFP–MCPH1-S were decreased by FLAG–CDH1 but not by FLAG–CDC20, in agreement with our data for endogenous MCPH1 (Fig. 5*B*). Initially, we mutated the KEN-Box (KEN^mut^) in MCPH1-L and MCPH1-S and cotransfected with FLAG–CDH1. Surprisingly, we found that MCPH1-L–KEN^mut^ was still degraded by CDH1 (Fig. 5*C*, left-hand panel), whereas MCPH1-S–KEN^mut^ was not (Fig. 5*C*, right-hand panel). This finding suggests that the 2 major isoforms of MCPH1 are regulated in different ways by APC/C–CDH1. As we were performing these studies, a paper was published suggesting that D-Box4 was important for MCPH1-L degradation (23). In agreement, we found that mutation of this D-Box (RxxL to AxxA, DB4^mut^) blocked the CDH1-dependent degradation of MCPH1-L. To verify our findings of MCPH1 isoform-specific regulation, we performed cotransfection studies with either MCPH1-L, MCPH1-L–DB4^mut^, MCPH1-S, or MCPH1-S–KEN^mut^ with either FLAG, FLAG–CDH1, or FLAG–CDC20. As shown in Fig. 5*D*, the CDH1-dependent degradation of MCPH1-L required D-Box4, whereas the CDH1-dependent degradation of MCPH1-S required the KEN-Box; CDC20 did not cause the degradation of any MCPH1 variants.

### D-Box4 phosphorylation is not involved in CDH1-mediated regulation of MCPH1

**Figure 6:**
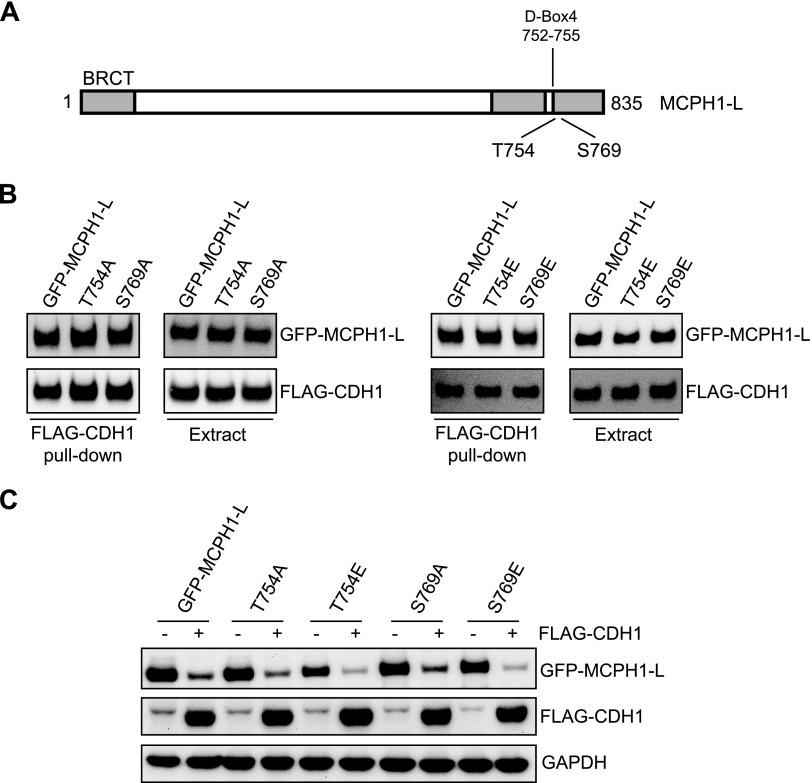
D-Box4 phosphorylation is not involved in CDH1-mediated regulation of MCPH1. *A*) Schematic diagram of MCPH1-L showing the positions of D-Box4 and the novel phosphorylation site in or near this degron sequence. *B*) GFP or GFP–MCPH1 variants were transfected into HEK293 cells for 24 h before purification and incubation with FLAG–CDH1 cell extracts (from transfected HEK293 cells). The ability of CDH1 to bind MCPH1 was investigated by using Western blotting. *C*) HeLa Tet-On cells were cotransfected with GFP–MCPH1-L (or the indicated phosphomutants/phosphomimetics) and either FLAG empty vector or FLAG–CDH1. GFP–MCPH1-L expression was induced by the addition of tetracycline 10 ng/ml. After 24 h, cells were lysed and samples analyzed by using Western blotting.

The mass spectrometry analysis of purified GFP–MCPH1-L identified several novel sites of phosphorylation. Interestingly, one of these sites (Thr754) was within D-Box4, the degron sequence required for the degradation of MCPH1-L. Another novel site (Ser769) was also identified a few amino acids downstream of D-Box4 (indicated in **Fig. 6*A***). We therefore investigated whether phosphorylation of Thr754 or Ser769 regulated either the MCPH1-L–CDH1 interaction or the CDH1-mediated degradation of MCPH1-L. Phosphomutant (T754A and S769A) and phosphomimetic (T753E and S769E) variants of GFP–MCPH1-L were produced and utilized in pull-down experiments with FLAG–CDH1. As shown in Fig. 6*B*, mutation of neither Thr754 nor Ser769 affected the interaction between MCPH1-L and CDH1. In addition, in coexpression studies with phosphomutants/phosphomimetics cotransfected with FLAG or FLAG–CDH1, all variants of MCPH1-L were degraded by CDH1 (Fig. 6*C*). Together, these data suggest that Thr754 and Ser769 phosphorylation are not involved in the MCPH1-L–CDH1 interaction or the CDH1-mediated regulation of MCPH1-L.

### MCPH1-L KEN-Box or D-Box4 are not involved in the chromatin condensation function of MCPH1

**Figure 7:**
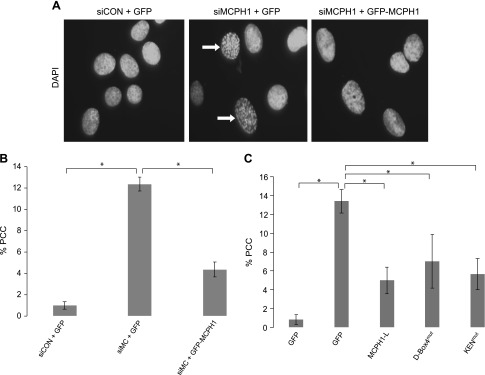
MCPH1-L KEN-Box or D-Box4 are not involved in the chromatin condensation function of MCPH1. *A*) U2OS cells were cotransfected with control siRNA (siCON) or MCPH1 siRNA (siMCPH1) along with either GFP empty vector or siRNA-resistant GFP–MCPH1-L. After 72 h, cells were fixed, stained with DAPI, and analyzed by fluorescence microscopy to identify PLCs that are characteristic of the PCC phenotype (indicated by white arrows). *B*) The percentage of PLCs was quantified after analyzing >300 cells. *C*) The studies outlined in *A* and *B* were repeated with the D-Box4 and KEN-Box mutant variants of MCPH1-L. The significance of the observed changes in PCC were investigated by using a Student’s paired *t* test. *P < 0.05.

A striking phenotype of MCPH1-deficient cells is the increased number of cells with prematurely condensed chromatin, often described as PLCs in the literature. Little is known about how this particular function of MCPH1 is regulated. Using siRNA, we efficiently depleted MCPH1 from U2OS cells and investigated the proportion of PLCs (indicated by the white arrow in **Fig. 7*A***) using fluorescence microscopy. Consistent with previous data, we found that control cells had <1% PLCs, whereas cells lacking MCPH1 had >12% PLCs. Importantly, GFP–MCPH1-L (with silent mutations in the siRNA target sequence) was able to partially correct this phenotype (Fig. 7*B*). The lack of full complementation is likely due to the transfection efficiency of GFP–MCPH1 being ∼70%. The lack of full complementation could also be due to the absence of endogenous MCPH1-S in the MCPH1 siRNA cells, with this isoform also able to correct the PLC phenotype in MCPH1-deficient cells (19). We next used the MCPH1-L–KEN^mut^ and MCPH1L–DB4^mut^ in complementation studies. As shown in Fig. 7*C*, all variants of MCPH1-L corrected the PCC phenotype to similar extents. Together, these data suggest that the KEN-Box and D-Box 4 (involved in the interaction with CDH1 and CDH1-dependent degradation, respectively) do not contribute to the chromatin condensation function of MCPH1.

## DISCUSSION

MCPH1 helps coordinate and control key events associated with mitotic entry, including CDK1 activation and chromosome condensation. In addition, MCPH1 functions as a tumor suppressor with roles in DNA damage repair, checkpoint activation, and apoptosis. Despite these roles, there is currently little information on how MCPH1 is regulated at the protein level. We found that both MCPH1 isoforms are phosphorylated during mitosis in a CDK1-dependent manner, followed by isoform-specific regulation by APC/C–CDH1.

To date, 14 genes have been shown to cause primary microcephaly when mutated. Interestingly, each of these genes has some role in mitotic regulation, but the PCC phenotype is unique to MCPH1 (24). Cell-free studies have suggested that MCPH1 is able to block the binding of condensin II to mitotic chromatin, helping maintain interphase chromatin in a noncondensed state (8). It follows that in a normal cell cycle, there must be a regulatory mechanism that alleviates the inhibitory effect of MCPH1 on condensin II, promoting chromosome condensation in mitosis. This mechanism does not involve the degradation of MCPH1, as we show that MCPH1 is present throughout mitosis and actually accumulates as cells are blocked in prometaphase, where chromatin is highly condensed. It is more likely that a rapid posttranslational modification of MCPH1 (or condensin II subunits) acts as a switch to help regulate MCPH1/condensin II–mediated chromatin condensation. Interestingly, we show that MCPH1 is phosphorylated in a CDK1-dependent manner in cells treated with nocodazole, and this phosphorylation is rapidly lost (within 10 min) as cells are released from the prometaphase block. It is tempting to speculate that mitotic phosphorylation of MCPH1 helps to maintain chromatin condensation by alleviating MCPH1-mediated inhibition of condensin II, and this scenario is reversed as MCPH1 is dephosphorylated during mitotic exit, allowing chromatin decondensation. In agreement, studies have shown that cells lacking MCPH1 have delayed chromosome decondensation at mitotic exit (5).

A recent study suggested that MCPH1-deficient cells undergo PCC in the G2 phase in a process dependent on CDK1 activity (9). This study showed that treatment with the CDK1 inhibitor (RO3306) for 4 or 7 h reduced the percentage of PLCs in MCPH1-deficient cell cultures by ∼10%. Other studies have also reported early activation of CDK1 (hypophosphorylation) in human and mouse cells lacking MCPH1 (3, 10). Although it is tempting to support a model whereby cells lacking MCPH1 undergo premature CDK1 activation, and this process drives condensin II–mediated chromosome condensation, such a model is difficult to reconcile with data showing lack of histone H3 Ser10 phosphorylation in PCC cells (3, 4), given that activation of CDK1 is the key event that triggers mitotic entry. It is clear that MCPH1 contributes to the regulation of chromosome condensation and CDK1 activation; how these events are coordinated and controlled, however, remains to be fully elucidated. To understand this process, it is essential to identify and characterize the cellular mechanisms that regulate the function of MCPH1 isoforms throughout the cell cycle.

There are limited studies investigating cell cycle–dependent regulation of MCPH1. Gavvovidis *et al.* (19) reported fluctuations in both MCPH1 isoforms at the transcript level, with MCPH1-L mRNA decreased from the mid-S phase to the G2 phase and decreased MCPH1-S mRNA in the early S phase before increasing in the late S/G2 phase. At the protein level, initial studies suggested that human MCPH1 was not regulated in a cell cycle–dependent manner, with no apparent change in protein levels or gel migration pattern in cell synchronization studies (17, 20). However, both studies used commercial MCPH1 antibodies (Ab2612, Abcam; and 4120, Cell Signaling Technology) that, in our validation studies, strongly recognized a nonspecific band at the approximate molecular mass of MCPH1-L. Hainline *et al.* (20) showed that Drosophila MCPH1 was degraded by APC/C–CDH1, but this effect was dependent on a D-Box present in an N-terminal extension of Drosophila MCPH1 that is completely absent in the human protein. This same study suggested that human MCPH1 was not degraded after release of HeLa cells from a nocodazole block, using the Cell Signaling Technology antibody. A recent study suggested that MCPH1 levels decreased in mitosis, before cyclin B1 degradation (23). Given that cyclin B1 is degraded by APC/C–CDC20 (activated before APC/C–CDH1), this observation would be inconsistent with MCPH1 being a target of CDH1. It is likely that the apparent decrease in MCPH1 in those studies was due to MCPH1 phosphorylation in mitosis and subsequent blocking of antibody binding, as shown in our validation studies using the MCPH1 antibody from Cell Signaling Technology. Unfortunately, we were unable to obtain precise information regarding the epitope used to generate this particular antibody, which could have allowed identification of the specific phosphorylation sites that block antibody binding. Cell Signaling Technology stated that this information was proprietary, although they did confirm that a “peptide surrounding Ser433 of human MCPH1 was used.” (personal communication). Our antibody testing results could be highly relevant when assessing the conclusions of some previously published data. In particular, care should be taken to ensure that the correct isoform of MCPH1 is being detected, and consideration should also be given to the potential effect of phosphorylation when using the Cell Signaling Technology antibody.

We found that both MCPH1 isoforms are degraded by APC/C–CDH1 after cells exit mitosis. CDH1 recognizes many of its target proteins *via* D-Box or KEN-Box degron sequences. We have described 2 such sequences in MCPH1-L that appear to be functionally relevant. The first sequence is a C-terminal D-Box that is necessary for CDH1-dependent degradation of MCPH1-L, in agreement with a recent study (23). The second is a KEN-Box sequence, mutation of which markedly reduced the interaction between MCPH1 and CDH1 but had no effect on CDH1-mediated degradation of MCPH1-L. It will be interesting to study the functional significance of this KEN-Box sequence in the large MCPH1 isoform. It is possible that MCPH1-L is also able to bind (*via* KEN-Box) and regulate the activity of APC/C–CDH1, or help recruit specific substrates *via* its 3 BRCT domains, which mediate protein–protein interactions. Another BRCT domain–containing protein, 53BP1, was recently shown to be both a target and regulator of APC/C–CDC20 (25). Interestingly, we have also shown that MCPH1-L is able to interact with CDC20 (data not shown), although CDC20 was not able to cause degradation of MCPH1-L or MCPH1-S.

In addition to controlling mitotic exit and G1 progression, APC/C–CDH1 contributes to DNA damage response pathways by degrading various proteins after ionizing radiation and UV radiation (26–28). After ionizing radiation, the resultant DNA double-strand breaks are repaired by either HRR (only in S/G2 phases) or non-homologous end joining (cell cycle independent). A recent study showed that CDH1 contributes to the choice of double-strand break repair pathway, promoting HRR by targeting the deubiquitinating enzyme USP1, which in turn allows recruitment of the pro-HRR BRCA1 to DNA double-strand breaks (29). Given that MCPH1 also positively contributes to HRR, it will be interesting to investigate whether the MCPH1-L KEN-Box (and thus MCPH1-L–CDH1 interaction) regulates this pathway. In contrast to MCPH1-L, the KEN-Box is required for CDH1-mediated degradation of MCPH1-S, showing that MCPH1 is degraded in an isoform-specific manner by APC/C–CDH1.

MCPH1 has an important role in brain development and has several hallmarks of a tumor suppressor protein, although the regulatory mechanisms that control MCPH1 function are poorly understood. We have shown that both MCPH1 isoforms are phosphorylated during mitosis in a CDK1-dependent manner and then regulated by APC/C–CDH1 in an isoform-specific manner. As well as clarifying how MCPH1 is regulated during the cell cycle, we are hopeful that the other regulatory mechanisms that we have identified, along with our observations with certain MCPH1 antibodies, will aid future research related to the important cellular roles of MCPH1.

## Abbreviations

APC/C: anaphase-promoting complex/cyclosome
BRCT: BRCA1 C-terminal domain
CDK1: cyclin-dependent kinase-1
CHK1: checkpoint kinase 1
GFP: green fluorescent protein
HRR: homologous recombination repair
MCPH1: microcephalin-1
MCPH1-L: long isoform of microcephalin-1
MCPH1-S: short isoform of microcephalin-1
MS/MS: tandem mass spectrometry
PCC: premature chromosome condensation
PLC: prophase-like cell
siRNA: small interfering RNA
